# The Acute Toxicity and Cardiotoxic Effects of Protocatechuic Aldehyde on Juvenile Zebrafish

**DOI:** 10.3390/toxics12110799

**Published:** 2024-11-03

**Authors:** Jiufeng Yin, Hui Wang, Feng Zhao, Dan Liang, Wenqing Yang, Dan Zhang

**Affiliations:** 1 Institute for Chinese Medicine Innovation, Shandong University of Traditional Chinese Medicine, Jinan 250300, China; yjf17854598221@163.com (J.Y.); m15192536410@163.com (H.W.); liangd980@163.com (D.L.); 2 Laboratory Centre, Shandong University of Traditional Chinese Medicine, Jinan 250300, China; 60019116@sdutcm.edu.cn; 3 Department of Classical Theory of Chinese Medicine, Key Laboratory of the Ministry of Education, Jinan 250355, China

**Keywords:** zebrafish, PCA, acute toxicity, cardiotoxic

## Abstract

Protocatechuic aldehyde (PCA) is a natural phenolic acid compound with pharmacological effects such as anti-oxidative stress, antibacterial, anti-apoptotic, anti-inflammatory, anti-platelet aggregation, and anti-tumor. Despite the favorable therapeutic effects of PCA, it is imperative to recognize that adverse drug reactions can arise even with satisfactory quality assurance measures and during standard clinical application and dosing. Additionally, the acute toxicity and cardiotoxic sequelae of PCA are frequently under reported in the available documentation. To investigate the acute toxicity and cardiotoxic effects of PCA, the present study comprehensively assessed the acute toxicity and cardiotoxic effects of PCA by administering different concentrations of PCA and by monitoring the phenotypic changes in zebrafish, using AB wild-type *Tg(cmlc2:EGFP)* zebrafish as the experimental model organism. Meanwhile, the target genes of PCA that may cause cardiotoxicity were predicted and validated using a network pharmacology approach. Our findings indicated that PCA exhibited severe acute toxicity and cardiotoxic effects in zebrafish at 70 μg/mL and 80 μg/mL. Furthermore, PIK3CA, PARP1, and GSK3β may be involved in the mechanism of action of the cardiotoxicity-inducing effects of this compound. The present investigation has afforded a deeper insight into the acute toxicity and cardiotoxic impacts of PCA on zebrafish and has established a significant theoretical foundation for the evaluation of toxicity in pharmaceuticals incorporating PCA.

## 1. Introduction

PCA is widely found in the roots of Salvia miltiorrhiza as well as in the leaves of the ophiopogon and hollyhock. It is a natural phenolic acid compound found in Salvia miltiorrhiza and other traditional Chinese medicinal herbs, and consists of a benzene ring and an acrolein moiety. Three hydroxyl groups are distributed on the benzene ring and the acrolein group is attached to the benzene ring, and this structure makes PCA hydrophilic and active [1,2]. Studies have shown that PCA possesses a variety of pharmacological activities such as antioxidant, antibacterial, anti-apoptotic, anti-inflammatory, anti-platelet aggregation, and anti-tumor, as well as many other pharmacological activities [3,4,5,6,7]. Currently, Cao found [8] that in rat animal experiments, PCA can be converted to protocatechuic acid in vivo, and the subsequent generation of vanillyl-CoA PCA has been a popular conjugate that inhibits the binding of long-chain fatty acids to CoA and reduces the AcCoA/CoA value [9,10,11,12]. 

In acute myocardial ischemia, the metabolism of PCA is slowed down, and the metabolism of protocatechuic acid and vanillic acid is slowed down in vivo, resulting in cardiotoxicity caused by accumulation [13]. This provides a rationale for the cardiotoxicity of the animal model in recent years for evaluating pharmaceutical effectiveness and safety. The zebrafish is a non-mammalian vertebrate known for its rapid development, tiny embryos and individuals, translucent body, high spawning rates, and rapid reproduction. The benefits of using zebrafish for experiments include reduced costs, shorter cycle times, and fewer ethical restrictions. It usually takes only about 3 days from the subject’s exposure to zebrafish to the completion of the evaluation experiment [14]. Furthermore, this study used transgenic fluorescently tagged zebrafish to reveal the labeled position under the corresponding excitation light and quantitatively analyze the area of cardiac fluorescence imaging. The zebrafish heart’s response to exogenous drugs is highly similar to that of human hearts [15]. This helps observe damage to organ shape and target the PCA medication. 

PCA’s cardiotoxic effects in zebrafish have not been documented in any national or international publication. We selected zebrafish as a new model organism for this study and administered different concentrations of the drug to zebrafish larvae to thoroughly assess the potential acute toxicity and cardiotoxicity of PCA exposure. This allowed us to provide important theoretical references for the evaluation of the toxicity of PCA drugs.

## 2. Materials and Methods

### 2.1. Instrumentation

A zebrafish aquaculture system (Beijing Aisheng Technology Co., Ltd., Beijing, China), Leica M205FA stereo fluorescence microscope (Leica Microsystems, Wetzlar, Germany), Zeiss LSM880 + Fast Airyscan Laser Confocal Microscope (with Live Cell Workstation), Leica RM2255 Paraffin Slicer, water bath LWB-24, pathological tissue processing staining system (YB-6LF, YR-19, ZT-12M), Gradient PCR Instrument T100, LongGene Real-Time Fluorescence PCR Instrument Q200B, Zebrafish Behavior Analyzer (ZebraLab), and ViewPoint Microscope ZEB6202 were used.

### 2.2. Medicines and Reagents

Procatechaldehyde (batch No. S30202-5g), xylene (batch No. W14278), hematoxylin anhydrous ethanol solution (batch No. R20587), and neutral gum (batch No. S30509) were acquired from Yuanye Biotechnology Co. (Shanghai, China). Tricine MS-222 (batch No. M14788) was bought from Jinan Anxia Biotechnology Co. Ltd, AbMole (Jinan, China). Fish fixative sodium carboxy methyl cellulose (CMC-Na, lot 419281) was bought from Sigma-Aldrich, St. Louis, MO, USA. Paraformaldehyde (batch No. CR2209049) was bought from Sevier (Wuhan, China). Biotechnology Co. 2xSYBR Green qPCR Mix (with ROX) (batch No. C13720975) was acquired from Ciscojet Biotechnology Co. A SPARKscript Ⅱ RT Plus Kit (with a gDNA eraser) (batch No. AG0304-B) was bought from Cisco Jie Biotechnology Co. (San Jose, CA, USA).

### 2.3. Animals

AB wild-type zebrafish *Tg(cmlc2:EGFP)* were acquired courtesy of Zebrafish Laboratory, New Animal Centre, Shandong University of Traditional Chinese Medicine, China. In a zebrafish culture system, every adult male and female zebrafish were kept apart and maintained at 28 °C, pH 7.2 ± 0.2, with a photoperiod of 14 h/10 h (light/dark) and twice-daily feedings of penaeid shrimp.

## 3. Methodology

### 3.1. Breeding and Culture of Zebrafish

Zebrafish reach sexual maturity at three months of age. Four to six pairs of mature fish were chosen from the tanks and put in a spawning device one night beforehand. The male and female fish were arranged in a 1:1 ratio, with an insert plate between them. To select high-quality embryos for zebrafish spawning, the lights were turned on and the transparent inserts were taken out the next morning. 

### 3.2. Zebrafish Drug Delivery Solution Preparation 

For this experiment, the PCA concentration gradient group (50 μg/mL, 60 μg/mL, 70 μg/mL, and 80 μg/mL) was chosen. One milligram of PCA was precisely weighed, put in a 1.5 mL EP tube, and dissolved in one milliliter of purified water to create a master batch with a drug mass concentration of one mg/mL. Next, 100 μL, 120 μL, 140 μL, and 160 μL of the masterbatch were taken from each tube and added to another 15 mL EP tube. Finally, 1900 μL, 1800 μL, 1600 μL, and 1400 μL of the masterbatch were added to the zebrafish embryo culture and diluted with water to the equivalent drug mass concentration of 50 μg/mL, 60 μg/mL, 70 μg/mL, and 80 μg/mL. The dispensed solution was diluted with water to an equivalent drug mass concentration of 50 μg/mL, 60 μg/mL, 70 μg/mL, and 80 μg/mL for further experimental drug delivery. The drug mass concentration drug delivery system was then formulated in each well of the total fish culture water of 2 mL in a 24-well plate. One liter of clean water included 0.633 g of KCl, 2.452 g of CaCl_2_, 14.658 g of NaCl, and 4.06 g of MgSO_4_·7H_2_O for zebrafish embryo culture. The anesthetic was a 0.4mg/mL tricaine solution prepared in purified water.

### 3.3. Grouping Interventions for Zebrafish

Five groups of experimental zebrafish per Section 2.3 were created: control and 50 μg/mL, 60 μg/mL, 70 μg/mL, and 80 μg/mL drug concentration groups for PCA. The drug concentration groups of 50 μg/mL, 60 μg/mL, 70 μg/mL, and 80 μg/mL PCA were immersed in drug preparation concentrations based on the experimental requirements, as determined by the method of Section 3.2. Meanwhile, the control group was maintained in zebrafish culture water without any intervention.

### 3.4. Zebrafish with Acute Exposure to PCA Exhibit Teratogenic Toxicity and Epimorphological Alterations

Normal zebrafish embryos were chosen under a somatic microscope upon spawning and placed onto 24-well plates, with ten embryos per well. The subjects had exposure to three duplicate wells in each of the following groups: the control group, the PCA concentration gradient group (50 μg/L, 60 μg/L, 70 μg/L, and 80 μg/mL), and soak-in drugs. To enable the embryos to continue developing, each well was filled to a total amount of 2 mL of embryo culture water, sealed, and kept in a fish room with a thermostat set at 28 °C. Per FET guidelines [16] and OECD guidelines [17], all developmental endpoints were identified. Every 24 h, the exposure solution was replaced at 24 hpf (hours after the hour after fertilization), 48 hpf, 72 hpf, and 96 hpf, respectively. The mortality of each group was recorded for four days in a row. Thirty zebrafish were fixed under a somatotopic microscope in 0.3% methylcellulose solution after being anesthetized with 0.4 mg/mL of tricaine as needed for observation. The zebrafish were then positioned sideways on slides with their eyes facing opposite directions to observe the embryos with aberrant development at various times. Every 24 h, hatchability and mortality were carefully tallied. At 96 h, the malformation rate was computed.

### 3.5. Acute Exposure to PCA’s Effects on Zebrafish Larvae’s Behavioral Alterations

Similar to the grouping under Section 3.3, zebrafish embryos subjected to a gradient concentration of 120 hpf PCA were placed in a Zebrabox, a Zebrafish ViewPoint Behaviore Analyzer, with one larval fish per well, on a 96-well plate. As soon as the analytical setup was complete, the behaviors of the zebrafish were investigated.

### 3.6. Acute Exposure to PCA and Its Impact on the Zebrafish Heart’s Sinus Venosus–Arteriolar Bulb (SV-BA) Distance

Zebrafish with 96hpf *Tg(cmlc2:EGFP)* were chosen and followed Section 3.3. Under a stereo microscope, thirty zebrafish were fixed in 0.3% methylcellulose solution after being anesthetized with 0.4 mg/mL tricaine. The fish were then placed sideways on slides across from their eyes, and the heart’s fluorescence was recorded and captured on camera. Confocal imaging analysis measured the area of PCA on the fluorescent heart, measured the distance between the sinus venous (SV) and the bulb of the artery (BA) to quantify the cardiac tube ring, and photographed the zebrafish fluorescent heart under excitation light [18].

### 3.7. Morphological Alterations in the Heart of Zebrafish Exposed to PCA 

In vivo imaging of the heart of zebrafish at the macroscopic and histological levels was observed under excitation light using the 96 hpf *Tg(cmlc2:EGFP)* zebrafish line of EGFP-labeled cardiomyocytes, as described in Section 3.3 Calculation of the effect of PCA on the fluorescence morphology area of the zebrafish heart at gradient concentrations and confocal imaging was carried out to analyze differences in cardiac morphology. The study used confocal imaging to examine variations in heart morphology.

### 3.8. Acute Exposure to PCA: Effects on the Heart Function of Zebrafish

The AB zebrafish were chosen and arranged in Section 3.3. Following a 96 h drug administration period, 30 zebrafish were anesthetized with 0.4 mg/mL tricaine, fixed in 0.3% methylcellulose solution under a somatotrope, and arranged sideways on slides with their eyes facing opposite directions. Heart rates were then counted every 20 s using the ViewPoint system’s photos and videos, which allow for the visual analysis of animal behavior. Zebrafish embryo heart rates were recorded at 20 s intervals in pictures and movies. To evaluate PCA-induced cardiotoxicity, the pericardial area was measured and drawn to determine the degree of pericardial edema [19].

### 3.9. The Impact of Brief Exposure to PCA on the Zebrafish Heart’s Histological Composition

After fixation with a gradient of ethanol, alcohol benzene, and xylene in an embedding machine in paraffin embedding, 4 μm sections were cut, deparaffinized to water, rinsed in pure water, and stained with hematoxylin–eosin (HE). The 96 hpf AB wild-type zebrafish, which are the same as the Section 3.3 grouping drug modeling for 96 h, were taken in the appropriate amount of 0.4 mg/mL tricaine anesthesia. Sections were cleaned, sealed with neutral gum, dried by air, and then put in gradient ethanol xylene and dehydrated until transparent before being photographed.

### 3.10. Prediction and Enrichment Analysis of Target Genes for PCA-Induced Cardiotoxicity

**Figure 1:**
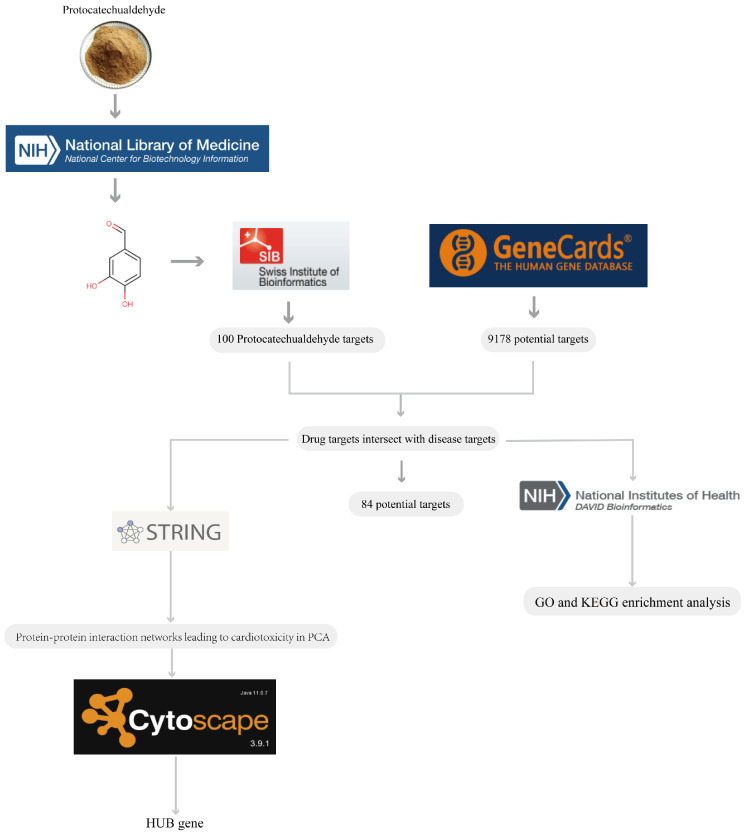
Schematic diagram of the network pharmacology process.

Using the SwissTarget website prediction (https://swisstargetprediction.ch, accessed on 1 January 2024) for PCA, possible target genes for the agent’s action were found. You may discover targets of cardiotoxic effects by searching for the word “cardiotoxicity” at https://www.genecards.org/Sitesearch, accessed on 1 January 2024. The 84 proteins that were obtained from SwissTarget and Gene Cards were entered into the String database (https://cn.string-db.org, accessed on 1 January 2024) to identify the corresponding zebrafish target genes. Utilizing the Microbiotics Online Analysis Network (https://www.bioinformatics.com.cn, accessed on 1 January 2024), the target genes identified by screening were imported. Figure 1 depicts the network pharmacological flow. The GO functional classification and KEGG enrichment analysis were performed based on the gene identifications for MF (molecular function), BP (biological process), and CC (cellular components). PIK3CA, PARP1, and GSK3β were identified as the main target genes of PCA cardiotoxicity using network pharmacogenomics. By using RT-PCR, this identity was verified. Using 96 hpf AB wild-type zebrafish (the same as Section 3.3), which were split into groups and immersed in the drug for 96 h, each group’s 50 zebrafish underwent two rounds of rinsing in pure water to remove any leftover water. The fish tissue was then extracted in its entirety to prepare the total RNA and transferred to 1.5 mL of non-enzymatic sterilization of EP tubes, and reverse transcription was carried out according to the instructions provided by the RNA Reverse Transcription Kit. The cDNA was reverse transcribed following the directions included with the RNA Reverse Transcription Kit, using β-actin as an internal reference throughout the PCR process. Reaction conditions: 95 °C for 3 min; 94 °C for 10 s; 65 °C for 30 s; 40 cycles. The 2^-ΔΔCt^ method was used to ascertain the mRNA’s relative expression. The primer sequences are shown in Table 1.

### 3.11. Statistical Analysis

All experiments were carried out at least three times, and all data are presented as mean ± S.D. Statistical significance and correlation between the groups were performed by SPSS 25.0 software (SPSS, Inc., Chicago, IL, USA). All data were analyzed with one-way analysis of variance (ANOVA) followed by Duncan’s multiple range tests. Values of *p* < 0.05 were considered statistically significant.

## 4. Results

### 4.1. Examination of the Morphological Alterations and Teratogenic Effects of Acute Exposure to PCA in Zebrafish

**Figure 2:**
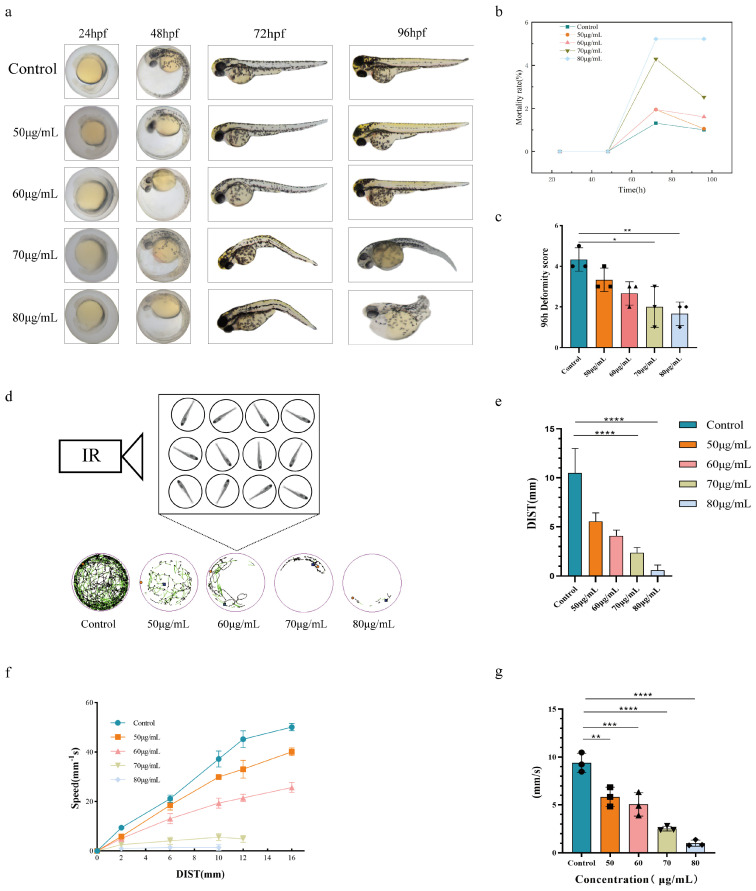
Acute toxic effects of PCA in zebrafish. (**a**) Single fish images of zebrafish larvae exposed to PCA gradient concentrations at 24 hpf, 48 hpf, 72 hpf, and 96 hpf (**b**) Mortality of zebrafish larvae exposed to PCA gradient concentrations at 96 hpf. (**c**) Deformity score of zebrafish larvae exposed to PCA gradient concentrations at 96 hpf. (**d**) Schematic diagram of the behavioral trajectory recordings of 120 hpf zebrafish at a gradient concentration of PCA under Zebrabox. (**e**) Statistical graph of distance analysis of the behavioral trajectory of 120 hpf zebrafish at a gradient concentration of PCA. (**f**) Statistical graph of the speed analysis of the behavioral trajectory of 120 hpf zebrafish at a gradient concentration of PCA. (**g**) Statistical graph of speed–distance analysis of the behavioral trajectory of 120 hpf zebrafish at a gradient concentration of PCA. Comparison with the blank control group: * *p* < 0.05; ** *p* < 0.01; *** *p* < 0.001; **** *p* < 0.001 (n = 30). The circles, squares, triangles, inverted triangles, and diamonds in the diagram represent the data in the histogram. Concentrations of PCA are expressed in μg/mL.

PCA gradient concentrations of 50 μg/mL, 60 μg/mL, 70 μg/mL, and 80 μg/mL were selected for observation and photographed at 24 hpf, 48 hpf, 72 hpf, and 96 hpf, respectively (Figure 2a–c). The figure shows that the zebrafish in the 60 μg/mL exposure group exhibited noticeable deformities at 72 h. Zebrafish showed spine curvature as the length of exposure to PCA increased. Zebrafish in the 60 μg/mL exposure group also showed swim bladder closure, loss, and pericardial edema in addition to yolk cysts. At 96 h, the zebrafish in the 80 μg/mL exposure group had the most prominent exhibition of the previously indicated phenomena, and their mortality rate significantly increased as the exposure concentration increased. This suggests that acute exposure to PCA affects zebrafish larval development in a concentration-dependent manner. 

### 4.2. An Examination of the Behavior of Zebrafish Larvae Exposed to PCA Acutely

Using a video monitoring and behavioral analysis system, the activity process of zebrafish exposed to different doses of PCA was recorded in this work. Before filming, the fish were allowed ten minutes to become used to their new environment. Behavioral data including speed, distance traveled, and duration of activity were then extracted from the video recordings. The overall decrease in the three previously indicated behavioral traits of the juvenile fish in the exposure group of 70 μg/mL is shown in Figure 2d–g. Conversely, in zebrafish at 120 hpf, the 80 μg/mL exposure group suppressed the mean rate and distance traveled and significantly reduced activity time and distance traveled. Therefore, PCA significantly reduced the zebrafish larvae’s ability to move their locomotor system at dosages of 70 μg/mL and 80 μg/mL. Meanwhile, PCA led to behavioral limitations in zebrafish, indicating the occurrence of some neurotoxicity.

### 4.3. Evaluation of the Acute Exposure of Zebrafish Larvae Heart SV-BA Distance to PCA

**Figure 3:**
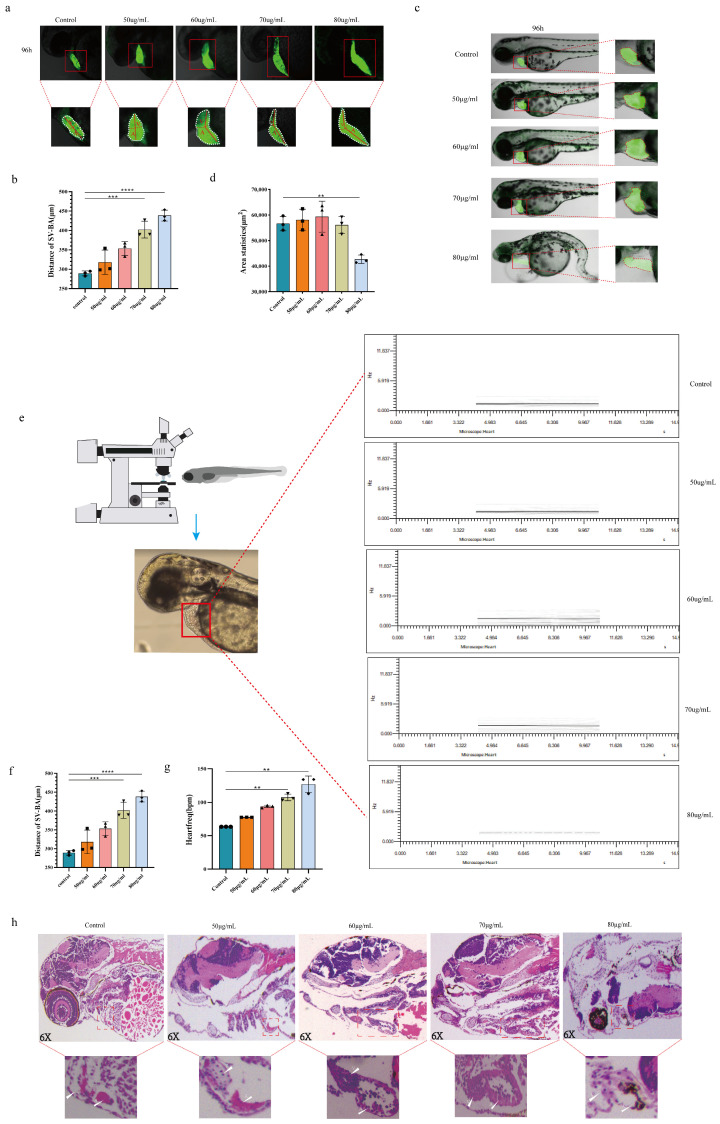
Cardiotoxicity effects of PCA in *Tg(cmlc2:EGFP)* zebrafish. (**a**) Confocal imaging of *Tg(cmlc2: EGFP)*: The control group and PCA gradient concentration group zebrafish larvae SV-BA distances in bright and fluorescent fields of view. (**b**) Statistical analysis graph (SV-BA distance). (**c**) Morphology of zebrafish larvae heart in bright and fluorescent fields of view in the confocal imaging: the control group and PCA gradient concentration group of *Tg(cmlc2:EGFP)*. (**d**) Graph of statistical analysis of the fluorescence area of the heart. (**e**) Schematic diagram of zebrafish heartbeat frequency observed under the ViewPoint system. (**f**) Statistical graph of the heartbeat frequency (HZ). (**g**) Statistical graph of the heartbeat frequency (bpm). (**h**) Effect of PCA gradient concentration on the histopathological structure of the heart of zebrafish larvae. White arrows represent V-A: ventricle–atrium. Note: comparison with blank control group: ** *p* < 0.01; *** *p* < 0.001; **** *p* < 0.001(n = 30). The circles, squares, triangles, inverted triangles, and diamonds in the diagram represent the data in the histogram.

Zebrafish cardiac SV-BA distance may be used to gauge the extent of heart injury. A decrease in the heart stroke volume, per pulse output, ejection fraction, and short axis shortening rate, is linked to an increase in this distance [20]. Thus, the SV-BA distance was the main indication used in this experiment to evaluate the toxicity of PCA to zebrafish. The effects of gradient PCA concentrations at 50 μg/mL, 60 μg/mL, 70 μg/mL, and 80 μg/mL on morphological changes in the *Tg(cmlc2:EGFP)* heart after 96 h were seen using a confocal imaging system. As compared to the control group, Figure 3a,b shows that the 80 μg/mL exposed group saw an increase in pericardial enlargement and SV-BA distance (*p* < 0.01). There was a discernible decrease in cardiac output and output per beat, along with a significant delay in the zebrafish yolk sac’s absorption, in the 70 μg/mL and 80 μg/mL exposure groups. It has been shown that the drug PCA may induce severe cardiotoxicity in zebrafish larvae at doses of 70 μg/mL and 80 μg/mL. 

### 4.4. Examination of the Morphological Alterations in the Heart of Zebrafish Exposed to PCA

Zebrafish cardiotoxicity from PCA may be seen right away in the atypical changes in their cardiac anatomy. Therefore, after 96 h, experiments were conducted using confocal imaging equipment to determine the effects of gradient concentrations of PCA of 50 μg/mL, 60 μg/mL, 70 μg/mL, and 80 μg/mL on the changes in heart shape of *Tg(cmlc2: EGFP)*. Figure 3c,d show that the zebrafish heart shape was not altered substantially in the 50 μg/mL and 60 μg/mL exposure groups compared to the control group. On the other hand, the zebrafish heart morphology in the 70 μg/mL exposure group exhibited longitudinal malformation, whereas the heart ventricle in the 80 μg/mL exposure group had distinct alterations, such as pericardial edema and longitudinal deformity of the atria and ventricles. According to these results, cardiac damage in PCA was seen at concentration of 70 μg/mL and 80 μg/mL. This implies that cardiac damage occurred at 70 μg/mL and 80 μg/mL of PCA.

### 4.5. Evaluation of Zebrafish Heart Function after Acute PCA Exposure

An excessive heart rate causes the coronary artery flow to decrease and the myocardial diastolic period to shorten, results in inadequate filling, and even causes the compensatory significance to disappear because of the decrease in output per minute. Blood enters the zebrafish heart via the venous sinus and leaves the ventricle through the arterial bulb. Since the zebrafish heart rate reflects the heart’s pumping function [21], it is employed as a supplemental indicator to assess the zebrafish heart function. The effects of gradient concentrations of PCA at 50 μg/mL, 60 μg/mL, 70 μg/mL, and 80 μg/mL on changes in zebrafish larval heart function at 96 hpf were identified using the Zebrafish ViewPoint system. As shown in Figure 3e, a deeper black line denotes a regular and powerful heartbeat, whereas a lighter black line with short intervals suggests a rapid, feeble, and irregular heartbeat. As shown in Figure 3f,g, compared to the control group, the heartbeat frequencies of the exposed groups at 70 μg/mL and 80 μg/mL were weak, rapid, irregular, and dysfunctional. The phenotypic traits were also more prominent in the 80 μg/mL exposed group. At concentrations of 70 μg/mL and 80 μg/mL, PCA was shown to affect zebrafish heart function, leading to abnormalities in heart rate and rhythm, cardiac insufficiency, and a reduction in the pumping capacity.

### 4.6. Analysis of the Histopathological Slice of the Heart of a Zebrafish Exposed to PCA

After 96 h, HE staining was used to investigate the effects of gradient concentrations of PCA (50 μg/mL, 60 μg/mL, 70 μg/mL, and 80 μg/mL) on histopathological alterations in the heart of zebrafish larvae. As per Figure 3h, there was no discernible difference between the 50 μg/mL and 60 μg/mL exposure groups and the control group in the histopathological sections of the heart. Conversely, the zebrafish heart tissues subjected to 70 μg/mL and 80 μg/mL had pronounced intercellular vacuoles, damaged nuclei, inflammatory infiltration, and hazy cell-to-cell borders, indicating serious structural abnormalities and impairments in the heart cellular structure. 

### 4.7. Cardiotoxic Target Gene Prediction and Enrichment Analysis Triggered by Acute PCA Exposure

#### 4.7.1. Potential Targets for PCA-Induced Cardiotoxicity

**Figure 4:**
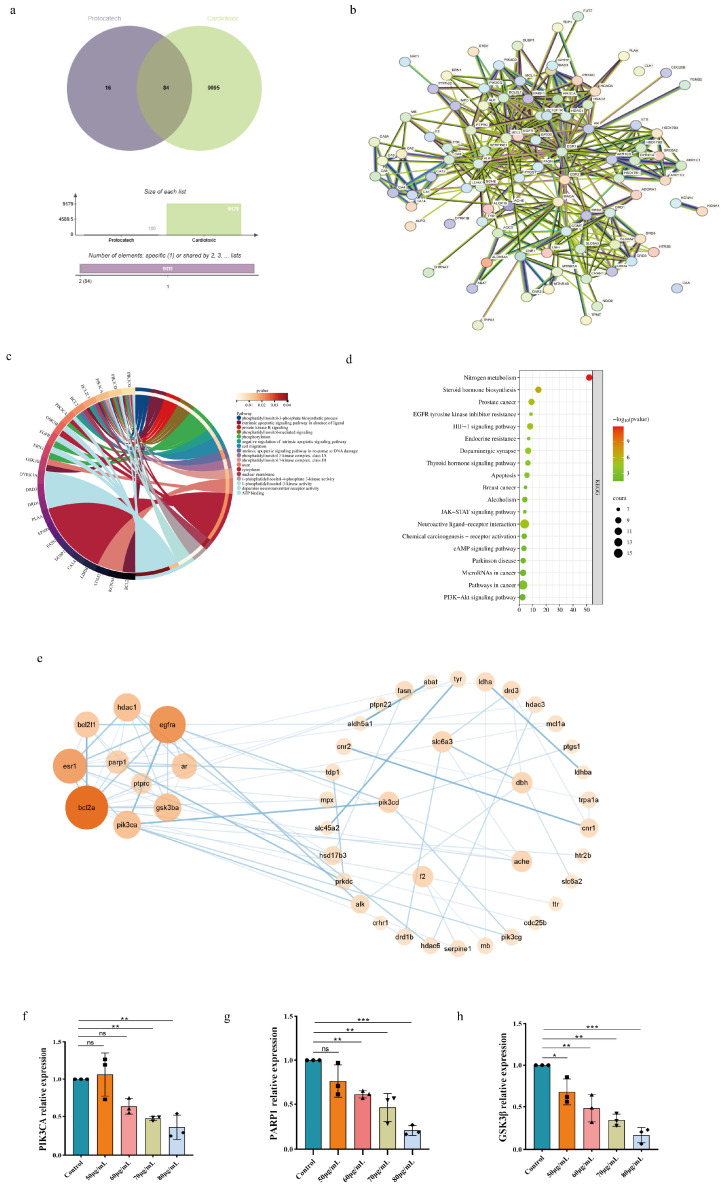
Network pharmacology chart. (**a**) Wayne diagram of cardiotoxicity and PCA intersection genes. (**b**) PPI network diagram. (**c**) GO analysis chart. (**d**) KEGG analysis chart. (**e**) Cytoscape HUB gene map. (**f**–**h**) Effect of PCA on PIK3CA, PARP1, and GSK3β mRNA expression of key target genes of zebrafish cardiotoxicity. Note: comparison with blank control group: * *p* < 0.05; ** *p* < 0.01; *** *p* < 0.001 (n = 50). The circles, squares, triangles, inverted triangles, and diamonds in the diagram represent the data in the histogram.

The SwissTarget database was used to determine the 100 active component targets of PCA. The active ingredient targets were retrieved from the SwissTarget database, and 100 drug targets were acquired after duplicates were removed. As shown in Figure 4a, 9179 cardiotoxic illness targets were retrieved using GeneCards, and 84 common targets were produced by crossing drug targets with disease targets. These common targets are probable targets of cardiotoxicity induced by PCA.

#### 4.7.2. Networks of Protein Interactions

Figure 4b shows the PPI network of possible targets of cardiotoxicity induced by PCA dispersions by (https://string-db.org/, accessed on 1 January 2024). The top 10 target genes of PIK3CA, PARP1, HDAC1, GSK3β, EGFRA, ESR1, BAL211, BCL2A, AR, and PTPRC were selected based on the degree of significance targeting values by using Cytoscape 3.9.1 software, as shown in Figure 4e.

#### 4.7.3. GO and KEGG Pathway Enrichment Analysis Results

To investigate the potential cardiotoxicity mechanisms of PCA, we used network pharmacology to predict the target genes that PCA might act on and performed KEGG and GO functional enrichment analyses of these target genes. As shown in Table 2, the target genes were found to be enriched in multiple pathways, including “phosphorylation, phosphatidyli-n-ositol-3-phosphatebiosynthesis, phosphatidylinositol-mediated, signaling, cytoplasmic, axonal, phosphatidylinositol-3-kinase, complexclasslA, ATPbinding, 1-phosphatidylinositol-4-ph-osp-hate, 1-phosphatidylinositol-3-kinase, or phosphate action”. Entries were filtered by *p* ≤ 0.05 and plotted as an enrichment analysis circle diagram in Figure 4c. We found that PCA may act on zebrafish cardiotoxicity through biological processes. The results of KEGG analysis showed that these biological processes mainly include “ErbB signaling pathway, apoptosis and inositol phosphate metabolism” (Table 3, Figure 4d). Combining the results of the enrichment analyses, we found that the mechanism of action of PCA-induced cardiotoxicity in zebrafish may be related to the core targets of PIK3CA, PARP 1, HDAC 1, GSK3β, EGFRA, ESR 1, BAL211, BCL 2A, AR, and PTPRC. Based on the existing reports, we selected three key target genes (PIK3CA, PARP1, and GSK3β), which are closely related to cardiotoxicity, from which we performed mRNA validation.

#### 4.7.4. Acute Exposure to PCA’s Effects on the mRNA Expression of the Main Target Genes for Cardiotoxicity in Zebrafish

We exposed zebrafish larvae to gradient concentrations of PCA (50 μg/mL, 60 μg/mL, 70 μg/mL, and 80 μg/mL) for 96 h. RT-PCR validation revealed a decreasing trend in the expression of PIK3CA, PARP1, and GSK3β in the gradient concentrations of PCA with the increase in drug concentration. As shown in Figure 4f, compared with the control, at PCA concentrations of 50 μg/mL and 60 μg/mL, there was no significant change in the mRNA expression of the PIk3CA gene. However, at 60 μg/mL and 70 μg/mL, the expression of the gene PIk3CA showed a decreasing trend compared with the control. There was no significant change in the mRNA expression of the PARP1 gene at 50 μg/mL PCA concentration compared with the control group, but the expression showed a decreasing trend at 60 μg/mL, 70 μg/mL, and 80 μg/mL, as shown in Figure 4g. As shown in Figure 4h, the mRNA expression of gene GSK3β decreased with the increase in PCA concentration, and the difference was statistically significant. This suggests that PCA may cause cardiotoxicity by inducing the expression of PIK3CA, PARP1, and GSK3β, especially the GSK3β gene; however, further systematic validation is needed to illustrate this.

## 5. Discussion

PCA has a wide range of pharmacological activities, and its pharmacological effects have been mainly focused on cardioprotective effects [22]. Several studies have reported that acute/chronic exposure to PCA may lead to toxic effects. In the present study, we used zebrafish as a sensitive and susceptible biological model for evaluating the toxicity of PCA drugs. Acute toxicity in zebrafish embryos is commonly manifested as behavioral changes such as the closure of the swim bladder, yolk cysts, loss of swim bladder, bending of the zebrafish trunk, and abnormal motor trajectories, suggesting the manifestation of neurotoxicity [23,24]. Pericardial edema and abnormal ring formation are frequently observed in zebrafish embryos as morphological changes of cardiac defects [25,26], and these changes can be reported on the basis of quantification of the pericardial area and SV-BA distance [27,28]. In addition, the most common dysfunction of the zebrafish cardiovascular system is abnormal heart rhythms, including tachycardia, bradycardia, atrioventricular block, premature beats, or fibrillation [29]. Miao Wenyu [30] showed that the Pearson correlation coefficient between zebrafish embryonic LC50 values and rodent LD50 values was 0.9271, showing a very strong positive correlation, and a one-way regression was fitted to the equation y = 0.4525x. Combined with previous studies [22], the LD50 of PCA in rodents was 1627 ± 115 mg/kg. The LD50 equation was used to calculate the LD50 of PCA on zebrafish as 57.297 ± 5.02 μg/mL. In addition, in an attempt to further investigate the degree of toxicity of PCA to zebrafish at the lethal concentration, we combined the pre-test with a simultaneous selection of concentrations exceeding the upper limit of the PCA (LD50) concentration to observe the maximal toxicity of PCA to zebrafish. On this basis, the doses of 50 μg/mL, 60 μg/mL, 70 μg/mL, and 80 μg/mL were selected for the phenotypic observation of 96 hpf zebrafish. At these dose-induced doses, we observed severe acute toxic injury and cardiac morphological changes in zebrafish, including pericardial edema, structural deformities, altered cardiac function, and pathological structural damage. Based on this, in the present study, we observed the acute toxicity of wild-type zebrafish by counting the acute toxicity after the administration of a gradient concentration of PCA, including observation of the overall morphological deformity changes, and monitoring the behavioral ability and cardiotoxicity by observing the cardiotoxicity in *Tg(cmlc2: EGFP)* zebrafish, including the area of cardiac fluorescence, cardiac function, and cardiac pathology slides for comprehensive evaluation of the intensity of the acute and cardiotoxicity of the gradient concentration of PCA. It was found that the concentration of PCA at 70 μg/mL resulted in irregular growth patterns, restricted behavioral function, led to cardiac malformation, and caused abnormal cardiac function in zebrafish, and this abnormality increased with the increase in exposure concentration. In order to investigate the locomotor behavior of zebrafish under acute exposure to PCA, an autonomous locomotion experiment was conducted in this study, and the results showed that zebrafish larvae exposed to PCA at the concentrations of 70 μg/mL and 80 μg/mL showed a significant decrease in the distance and speed of locomotion. These abnormal behaviors indicated that the exposure to PCA at 70 μg/mL and 80 μg/mL resulted in acute toxicity and abnormal behavioral functions in zebrafish. Based on this, the present study was the first to comprehensively and systematically evaluate the effects of PCA on acute toxicity and cardiotoxicity and to preliminarily elucidate the mechanism of action of PCA-induced cardiotoxicity using a combination of phenotypic observation, network pharmacology, and experimental validation using zebrafish of the AB and *Tg(cmlc2:EGFP)* strains. It provides an important theoretical reference for the evaluation of the drug toxicity of PCA.

In the follow-up experiments, combined with enrichment analysis, three key genes, PIK3CA, PARP1, and GSK3β, were selected. Among the key target genes, we enriched the KEGG pathway and further analyzed it by combining it with the existing literature. The PI3K-AKT signaling pathway in the signaling pathway enrichment results has been proven to be related to a variety of cardiovascular diseases [31,32,33,34]. The phosphatidylinositol-45-bisphosphate 3-kinase catalytic subunit alpha PIK3CA gene consists of multiple exons and is expressed in a variety of tissues and organs, and is widely involved in cell proliferation, survival, and cell cycle regulation, as well as other cellular functional activities [35]. Studies have shown that PIK3CA can activate PI3K, mediate the phosphorylation of Akt, and then regulate mTOR and GSK3β, which are involved in the regulation of cell proliferation, differentiation, and apoptosis [36,37,38]. PIK3CA is a key initiator of the PI3K-AKT signaling pathway, which has an important role in regulating biological signaling and pathway activation. He Yazhou [39] found that miR-320a could promote myocardial fibrosis through its target gene PIK3CA in protein interaction analysis of PI3K-AKT pathway target genes. PARP-1 (poly-ADP-ribose polymerase-1) is an important member of the PARP family whose main role is DNA damage repair and regulation of apoptosis [40]. Some studies have found that single nucleotide polymorphism changes in PARP-1 are associated with genetic susceptibility to many diseases [41]. Rumei Men [42] found that metoprolol reduces cardiomyocyte apoptosis and thus ameliorates MI/R injury mainly by inhibiting PARP-1 protein expression levels. GSK3β (glycogen synthase kinase 3β) is a class of highly structurally conserved serothreonine protein kinases that are prevalent in organisms and eukaryotes and play an important role in the regulation of cellular functions, such as cellular structure regulation, intracellular signaling, cell division, apoptosis, microtubule movement, and determination of the fate of cells in the process of embryonic development [43,44,45,46,47]. In recent years, many studies have confirmed that GSK3β is an important factor in the regulation of apoptosis [48]. Some studies have confirmed that the PI3K/AKT/GSK3β pathway promotes fibrosis in myocardial tissues, and its upregulation damages cardiomyocytes and participates in the pathogenesis of acute myocardial infarction [49,50,51]. Huang Jing [52] found that downregulation of miR-208 could inhibit PI3K/AKt/GSK3β signaling pathway-related proteins, which in turn inhibited cardiomyocyte apoptosis, alleviated the body’s inflammatory response, protected the body’s cardiomyocytes, and ultimately slowed down the degree of cardiac muscle damage. Combined with the above studies, it is hypothesized that the three key target genes, PIK3CA, PARP1, and GSK3β, are all associated with cardiomyocyte apoptosis. Apoptosis is a form of programmed cell death regulated by the exogenous death receptor pathway and the endogenous mitochondrial pathway, and it is essential for the normal development of the cardiovascular system in zebrafish [53]. However, the relevance of PIK3CA, PARP1, and GSK3β to PCA and their regulatory mechanisms to induce cardiotoxicity in PCA have rarely been reported [54,55]. Therefore, we performed RT-PCR to validate the expression of PIK3CA, PARP1, and GSK3β and found that the expression of PIK3CA, PARP1, and GSK3β decreased in a concentration-dependent manner with the PCA concentration gradient. It can be preliminarily speculated that the mechanism of PCA-induced cardiotoxicity may be related to the apoptosis of cardiomyocytes caused by the three core target genes, PIK3CA, PARP1, and GSK3β. This still needs further investigation and research.

In this experiment, we found that PCA produced severe acute toxicity and cardiotoxic effects in zebrafish at concentrations of 70 μg/mL and 80 μg/mL. In addition, PIK3CA, PARP1, and GSK3β may be involved in the mechanism of action of this compound to induce cardiotoxic effects. In addition, more biological model experiments are needed to help avoid the toxic side effects and improve the safety of drug use.

## 6. Conclusions

For the first time, we observed the acute toxicity and cardiotoxic effects of PCA in wild-type and transgenic zebrafish models and initially explored the mechanism of toxicity of PCA, with the aim of better understanding the potential toxicity of PCA in order to achieve the purpose of reducing the adverse effects of PCA and enhancing the medicinal value of PCA, to provide strong evidence for accelerating the development of new highly efficient and low-toxicity drugs containing PCA, and also to provide a valuable theoretical framework for assessing the toxicity of PCA containing drugs. This study provides strong evidence for accelerating the development of new efficient and low-toxicity drugs for PCA and provides a valuable theoretical framework for assessing the toxicity of PCA-containing drugs.

## Figures and Tables

**Table 1:** Primer sequences (5′–3′).

β-Actin	F: AGAGCTATGAGCTGCCTGACG	R: CCGCAAGATTCCATACCCA
Pik3ca	F: GATCGCCGAAGCCATCAGGAAG	R: GTCACAGCCGCAGACCTTCAG
Parp1	F: CATTTGGGTCCCTGAAGCCT	R: ACCCAGTCTTTGCGATCAGG
Gsk3β	F: AACTCTGCGACTTTGGCAGT	R:CGGTGGCTCCAAAGATGAGT

**Table 2:** GO enrichment analysis including molecular function (MF), cellular composition (CC), and biological process (BP).

GO Term	Subgroup	Count
Phosphatidylinositol-3-phosphate biosynthetic process	Biological Processes	3
Extrinsic apoptotic signaling pathway in the absence of ligand	Biological Processes	3
Protein kinase B signaling	Biological Processes	3
Phosphatidylinositol-mediated signaling	Biological Processes	3
Phosphorylation	Biological Processes	5
Negative regulation of intrinsic apoptotic signaling pathway	Biological Processes	2
Cell migration	Biological Processes	3
Intrinsic apoptotic signaling pathway in response to DNA damage	Biological Processes	2
Phosphatidylinositol 3-kinase complex, class lA	Cellular Components	2
Phosphatidylinositol3-kinase complex, class lB	Cellular Components	2
axon	Cellular Components	3
Cytoplasm	Cellular Components	10
Nuclear membrane	Cellular Components	2
1-Phosphatidylinositol-4-phosphate 3-kinase activity	Molecular Functions	3
1-Phosphatidylinositol-3-kinase activity	Molecular Functions	3
Dopamine neurotransmitter receptor activity	Molecular Functions	2
ATP binding	Molecular Functions	7

**Table 3:** Analysis of KEGG enrichment.

Number	Pathway	Enrichment	*p*-Value	Count
zab00140	ErbB signaling pathway	26.31292517	0.000364195	4
zab04210	Apoptosis	10.96371882	0.000778384	5
zab04140	Inositol phosphate metabolism	8.05028305	0.002457148	5
zab04012	FoxO signaling pathway	12.44530245	0.003254212	4
zab04510	Autophagy-animal	6.692967885	0.004793276	5
zab04068	Insulin signaling pathway	8.372294372	0.009852925	4
zab04910	Herpes simplex virus 1 infection	7.804681195	0.011931379	4
zab01100	Salmonella infection	2.030969565	0.017097321	11
zab05168	Human cytomegalovirus infection	6.771708683	0.017480242	4
zab00562	Metabolic pathways	10.62637363	0.028938037	3

## Data Availability

The raw data supporting the conclusions of this article will be made available by the authors upon request.

## References

[1] Zhang C., Guo L., Wang J. (2013). Progress of pharmacological studies on protocatechuic aldehyde. Chin. J. Exp. Formulas.

[2] Ji G.L., Zhou W., Ba R.R., Lin C.H., Liu Y.L. (2023). Determination of six phenolic acid components in Danshen formula granules by double-labeled multi-assay method. J. Pharm. Anal..

[3] Krzysztoforska K., Mirowska-Guzel D., Widy-Tyszkiewicz E. (2019). Pharmacological effects of protocatechuic acid and its therapeutic potential in neurodegenerative diseases: Review on the basis of in vitro and in vivo studies in rodents and humans. Nutr. Neurosci..

[4] Xing Y.L., Zhou Z., Agula, Zhong Z.Y., Ma Y.J., Zhao Y.L., Xiao X.H., Wang S.Q. (2012). Protocatechuic aldehyde inhibits lipopolysaccharide-induced human umbilical vein endothelial cell apoptosis via regulation of caspase-3. Phytother. Res..

[5] Fang X., Liu Y., Lu J., Hong H., Yuan J., Zhang Y., Wang P., Liu P., Ye J. (2018). Protocatechuic aldehyde protects against isoproterenol-induced cardiac hypertrophy via inhibition of the JAK2/STAT3 signaling pathway. Naunyn-Schmiedeberg’s Arch. Pharmacol..

[6] Wan Y.J., Guo Q., Liu D., Jiang Y., Zeng K.W., Tu P.F. (2019). Protocatechualdehyde reduces myocardial fibrosis by directly targeting conformational dynamics of collagen. Eur. J. Pharmacol..

[7] Yang S.Y., Xiao Y., An C., Qiu C.L., Li J.A., Chu J.X. (2024). Neuroprotective mechanism of protocatechuic aldehyde. Chin. Herbal. Med..

[8] Cao Y.G., Zhang L., Ma C., Chang B.B., Chen Y.C., Tang Y.Q., Liu X.D., Liu X.Q. (2009). Metabolism of protocatechuic acid influences fatty acid oxidation in rat heart: New anti-angina mechanism implication. Biochem. Pharmacol..

[9] Zhou J., Zhang J., Cao Y., Chen Y., Yu D., Liu X. (2009). Effect of acute myocardial ischemia on methylation metabolism of danshenin in rats. J. China Pharm. Univ..

[10] Zhu B.T. (2002). Catechol-O-Methyltransferase (COMT)-mediated methylation metabolism of endogenous bioactive catechols and modulation by endobiotics and xenobiotics: Importance in pathophysiology and pathogenesis. Curr. Drug Metab.

[11] Xu M., Zhang Z., Fu G., Sun S., Sun J., Yang M., Liu A., Han J., Guo D. (2007). Liquid chromatography-tandem mass spectrometry analysis of protocatechuic aldehyde and its phase I and II metabolites in rat. J. Chromatogr. B Anal. Technol. Biomed. Life Sci..

[12] Cao W., Cao Y., Zhang L., Chang Z., Tang Y., Liu X. (2010). Effects of acute myocardial ischemia on the pharmacokinetics of protocatechuic acid in rats. J. China Pharm. Univ..

[13] Gao L., Wu W.F., Dong L., Ren G.L., Li H.D., Yang Q., Li X.F., Xu T., Li Z., Wu B.M. (2016). Protocatechuic Aldehyde Attenuates Cisplatin-Induced Acute Kidney Injury by Suppressing Nox-Mediated Oxidative Stress and Renal Inflammation. Front. Pharmacol..

[14] MacRae C.A., Peterson R.T. (2015). Zebrafish as tools for drug discovery. Nat. Rev. Drug Discov..

[15] OECD (2012). Validation Report (Phase 2) for the Zebrafish Embryo Toxicity Test I.

[16] OECD (2013). Test No. 236: Fish Embryo Acute Toxicity (FET) Test.

[17] Huang M., Jiao J., Wang J., Xia Z., Zhang Y. (2018). Exposure to acrylamide induces cardiac developmental toxicity in zebrafish during cardiogenesis. Environ. Pollut..

[18] Liu H., Chu T., Chen L., Gui W., Zhu G. (2017). In vivo cardiovascular toxicity induced by acetochlor in zebrafish larvae. Chemosphere.

[19] Xue D. (2016). Construction and Study of Vasopressor and Cardiac Injury Model in Zebrafish. Master’s Thesis.

[20] Berman N., Lectura M., Thurman J., Reinecke J., Raff A.C., Melamed M.L., Reinecke J., Quan Z., Evans T., Meyer T.W. (2013). A zebrafish model for uremic toxicity: Role of the complement pathway. Blood Purif..

[21] Brown M.A., Magee L.A., Kenny L.C., Karumanchi S.A., McCarthy F.P., Saito S., Hall D.R., Warren C.E., Adoyi G., Ishaku S. (2018). Hypertensive Disorders of Pregnancy: ISSHP Classification, Diagnosis, and Management Recommendations for International Practice. Hypertension.

[22] Yang Y., Cao J., Xu X., Xing W., Liu Z., Xu J., Shi Y., Wang M., Wang G., Yang J. (1979). Distribution, excretion and toxicity of protocatechualdehyde in animals. Jiangsu Med..

[23] Zhou S.Y., Chen J.P., Liu Z.D., Zhou J.R., Liu Y., Liu S.H., Tian C.W., Chen C.Q. (2021). Progress in the study of chemical composition and pharmacological effects of Yam bean root. Chin. Herb. Med..

[24] Jang S.M., Bae S.H., Choi W.K., Park J.B., Kim D., Min J.S., Yoo H., Kang M., Ryu K.H., Bae S.K. (2015). Pharmacokinetic properties of trifolirhizin, (-)-maackiain, (-)-isophorone and 2-(2,4-dihydroxy phenyl)-5,6-methylenedioxybenzofuran after intravenous and oral administration of Sophora tonkinensis extract in rats. Xenobiotica.

[25] Duan J., Hu H., Li Q., Jiang L., Zou Y., Wang Y., Sun Z. (2016). Combined toxicity of silica nanoparticles and methylmercury on the cardiovascular system in zebrafish (*Danio rerio*) embryos. Environ. Toxicol. Pharmacol..

[26] Duan J., Yu Y., Li Y., Li Y., Liu H., Jing L., Yang M., Wang J., Li C., Sun Z. (2016). Low-dose exposure of silica nanoparticles induces cardiac dysfunction via neutrophil-mediated inflammation and cardiac contraction in zebrafish embryos. Nanotoxicology.

[27] Han Y., Zhang J.P., Qian J.Q., Hu C.Q. (2015). Cardiotoxicity evaluation of anthracyclines in zebrafish (*Danio rerio*). J. Appl. Toxicol..

[28] Han Y., Li X., Yan M., Yang M., Wang S., Pan J., Li L., Tan J. (2019). Oxidative damage induces apoptosis and promotes calcification in disc cartilage endplate cell through ROS/MAPK/NF-κB pathway: Implications for disc degeneration. Biochem. Biophys. Res. Commun..

[29] Shen R., Yu Y., Lan R., Yu R., Yuan Z., Xia Z. (2019). The cardiovascular toxicity induced by high doses of gatifloxacin and ciprofloxacin in zebrafish. Environ. Pollut..

[30] Miao W., Zhu X., Xu Y., Jiang Y., Shao L. (2022). Derivation of dose conversion factors for zebrafish and rodents based on experimental data. Chin. Sci. Technol. J. Database (Full Text. Version) Nat. Sci..

[31] Rofaani E., Mardani M.W., Yutiana P.N., Amanda O., Darmawan N. (2024). Differentiation of mesenchymal stem cells into vascular endothelial cells in 3D culture: A mini-review. Mol. Biol. Rep..

[32] Hu Y.Y. (2013). PI3K/Akt/GSK-3β and Mitochondrial ATP-Sensitive Potassium Channels Mediate the Protective Effect of Procyanidins against Myocardial Ischemia-Reperfusion Injury. Ph.D. Thesis.

[33] Han J., Xuan J., Hu H., Chen Z. (2015). Relationship between the effects of hypericin preconditioning to attenuate myocardial ischemia-reperfusion injury in rats and the PI3K/Akt signalling pathway. Chin. J. Tradit. Chin. Med..

[34] Díaz R., Goyal A., Mehta S.R., Afzal R., Xavier D., Pais P., Chrolavicius S., Zhu J., Kazmi K., Liu L. (2007). Glucose-insulin-potassium therapy in patients with ST-segment elevation myocardial infarction. JAMA.

[35] Raghunath A., Perumal E. (2018). Analysis of Lethality and Malformations During Zebrafish (*Danio rerio*) Development. Methods Mol. Biol..

[36] Sun L., Cui K., Xing F., Liu X. (2020). Akt dependent adult hippocampal neurogenesis regulates the behavioral improvement of treadmill running to mice model of post-traumatic stress disorder. Behav. Brain Res..

[37] Vicent L., Cinca J., Vazquez-García R., Gonzalez-Juanatey J.R., Rivera M., Segovia J., Pascual-Figal D., Bover R., Worner F., Delgado-Jiménez J. (2019). Discharge treatment with angiotensin-converting enzyme inhibitor/angiotensin receptor blocker after a heart failure hospitalization is associated with a better prognosis irrespective of left ventricular ejection fraction. Intern. Med. J..

[38] McMurray J.J., Packer M., Desai A.S., Gong J., Lefkowitz M., Rizkala A.R., Rouleau J.L., Shi V.C., Solomon S.D., Swedberg K. (2014). Baseline characteristics and treatment of patients in prospective comparison of ARNI with ACEI to determine the impact on global mortality and morbidity in heart failure trial (PARADIGM-HF). Eur. J. Heart Fail..

[39] He Y. (2019). Study on Exosome-Derived Mir-320a Regulating PIK3CA against Myocardial Fibrosis in Chronic Heart Failure by Wenzhong Yiqi Fang. Master’s Thesis.

[40] Wu R.C., Ayhan A., Maeda D., Kim K.R., Clarke B.A., Shaw P., Chui M.H., Rosen B., Shih Ie M., Wang T.L. (2014). Frequent somatic mutations of the telomerase reverse transcriptase promoter in ovarian clear cell carcinoma but not in other major types of gynecological malignancy. J. Pathol..

[41] Embi N., Rylatt D.B., Cohen P. (1980). Glycogen synthase kinase-3 from rabbit skeletal muscle. Separation from cyclic-AMP-dependent protein kinase and phosphorylase kinase. Eur. J. Biochem..

[42] Men R., Wang Y., Zhang L., Men L., Lan W., Meng Q., Yu J. (2024). Mechanism of action of metoprolol combined with clopidogrel in mice with myocardial ischemia-reperfusion injury. West. Med..

[43] Fang X., Yu S.X., Lu Y., Bast R.C., Woodgett J.R., Mills G.B. (2000). Phosphorylation and inactivation of glycogen synthase kinase 3 by protein kinase A. Proc. Natl. Acad. Sci. USA.

[44] Zhu H., Zhang W., Zhao Y., Shu X., Wang W., Wang D., Yang Y., He Z., Wang X., Ying Y. (2018). GSK3β-mediated tau hyperphosphorylation triggers diabetic retinal neurodegeneration by disrupting synaptic and mitochondrial functions. Mol. Neurodegener..

[45] Takahashi-Yanaga F. (2018). Roles of Glycogen Synthase Kinase-3 (GSK-3) in Cardiac Development and Heart Disease. J. Uoeh.

[46] Zhou B.P., Deng J., Xia W., Xu J., Li Y.M., Gunduz M., Hung M.C. (2004). Dual regulation of Snail by GSK-3beta-mediated phosphorylation in control of epithelial-mesenchymal transition. Nat. Cell Biol..

[47] Götschel F., Kern C., Lang S., Sparna T., Markmann C., Schwager J., McNelly S., von Weizsäcker F., Laufer S., Hecht A. (2008). Inhibition of GSK3 differentially modulates NF-kappaB, CREB, AP-1, and beta-catenin signaling in hepatocytes, but fails to promote TNF-alpha-induced apoptosis. Exp. Cell Res..

[48] Li H., Zha Y., Du F., Liu J., Li X., Zhao X. (2020). Contributions of PARP-1 rs1136410 C>T polymorphism to the development of cancer. J. Cell Mol. Med..

[49] Deng S., Dai G., Chen S., Nie Z., Zhou J., Fang H., Peng H. (2019). Dexamethasone induces osteoblast apoptosis through ROS-PI3K/AKT/GSK3β signaling pathway. Biomed. Pharmacother..

[50] Wang X., Zou W., Li J., Cai G., Liu K., Jia K., Wang T., Peng Y. (2020). Mechanistic study on the myocardial protective effect of cardiostatin on MIRI rats based on PI3K/Akt/GSK-3β signaling pathway. J. Chin. Med..

[51] Li S. (2021). Study on the Protective Mechanism of Myocardial Ischemia-Reperfusion Injury via ALKBH5/GSK3β/mTOR Signaling Pathway by Quick-Acting Heart-Saving Pill. Master’s Thesis.

[52] Huang J., Lei Y., Hua X., Zhou H., Zhu X. (2022). The intervention of miR-208a based on down-regulation of PI3K/AKT/GSK3β signaling pathway in acute myocardial infarction model rats. Hebei Med..

[53] Sinha K., Das J., Pal P.B., Sil P.C. (2013). Oxidative stress: The mitochondria-dependent and mitochondria-independent pathways of apoptosis. Arch. Toxicol..

[54] Burgos-Aceves M.A., Cohen A., Smith Y., Faggio C. (2018). MicroRNAs and their role on fish oxidative stress during xenobiotic environmental exposures. Ecotoxicol. Environ. Saf..

[55] Burgos-Aceves M.A., Cohen A., Paolella G., Lepretti M., Smith Y., Faggio C., Lionetti L. (2018). Modulation of mitochondrial functions by xenobiotic-induced microRNA: From environmental sentinel organisms to mammals. Sci. Total Environ..

